# Effect of Anti-seizure Medications on Functional Anatomy of Language: A Perspective From Language Functional Magnetic Resonance Imaging

**DOI:** 10.3389/fnins.2021.787272

**Published:** 2022-02-24

**Authors:** Fenglai Xiao, Lorenzo Caciagli, Britta Wandschneider, Bhavini Joshi, Sjoerd B. Vos, Andrea Hill, Marian Galovic, Lili Long, Daichi Sone, Karin Trimmel, Josemir W. Sander, Dong Zhou, Pamela J. Thompson, Sallie Baxendale, John S. Duncan, Matthias J. Koepp

**Affiliations:** ^1^Department of Neurology, West China Hospital, Sichuan University, Chengdu, China; ^2^Department of Clinical and Experimental Epilepsy, UCL Queen Square Institute of Neurology, London, United Kingdom; ^3^Chalfont Centre for Epilepsy, Chalfont St. Peter, United Kingdom; ^4^Department of Bioengineering, University of Pennsylvania, Philadelphia, PA, United States; ^5^Department of Neurology, The Royal London Hospital, London, United Kingdom; ^6^UCL Centre for Medical Image Computing, London, United Kingdom; ^7^Department of Neuroradiology, UCL Queen Square Institute of Neurology, London, United Kingdom; ^8^Department of Neurology, Clinical Neuroscience Center, University Hospital Zürich, Zurich, Switzerland; ^9^Department of Neurology, Xiangya Hospital, Central South University, Changsha, China; ^10^Department of Neurology, Medical University of Vienna, Vienna, Austria; ^11^Stichting Epilepsie Instellingen Nederland, Heemstede, Netherlands

**Keywords:** epilepsy, language functional MRI, drug load, cognitive effect, polytherapy

## Abstract

**Background:**

In epilepsy, cognitive difficulties are common, partly a consequence of anti-seizure medications (ASM), and cognitive side-effects are often considered to be more disabling than seizures and significantly affect quality of life. Functional MRI during verbal fluency tasks demonstrated impaired frontal activation patterns and failed default mode network deactivation in people taking ASM with unfavourable cognitive profiles. The cognitive effect of ASMs given at different dosages in monotherapy, or in different combinations, remains to be determined.

**Methods:**

Here, we compared the effects of different drug loads on verbal fluency functional MRI (fMRI) in people (i) taking dual therapy of ASMs either considered to be associated with moderate (levetiracetam, lamotrigine, lacosamide, carbamazepine/oxcarbazepine, eslicarbazepine, valproic acid; *n* = 119, 56 females) or severe (topiramate, zonisamide) side-effects; *n* = 119, 56 females), (ii) taking moderate ASMs in either mono-, dual- or triple-therapy (60 subjects in each group), or (iii) taking different dosages of ASMs with moderate side-effect profiles (*n* = 180). “Drug load” was defined as a composite value of numbers and dosages of medications, normalised to account for the highest and lowest dose of each specific prescribed medication.

**Results:**

In people taking “moderate” ASMs (*n* = 119), we observed higher verbal-fluency related to left inferior frontal gyrus and right inferior parietal fMRI activations than in people taking “severe” ASMs (*n* = 119). Irrespective of the specific ASM, people on monotherapy (*n* = 60), showed greater frontal activations than people taking two (*n* = 60), or three ASMs (*n* = 60). People on two ASMs showed less default mode (precuneus) deactivation than those on monotherapy. In people treated with “moderate” ASMs (*n* = 180), increased drug load correlated with reduced activation of language-related regions and the right piriform cortex.

**Conclusion:**

Our study delineates the effects of polytherapy and high doses of ASMs when given in monotherapy on the functional anatomy of language. Irrespective of the cognitive profile of individual ASMs, each additional ASM results in additional alterations of cognitive activation patterns. Selection of ASMs with moderate cognitive side effects, and low doses of ASMs when given in polytherapy, could reduce the cognitive effect.

## Introduction

The ultimate goal of treatments with anti-seizure medications (ASM) for epilepsy is to prevent seizures without causing side effects. One-third of people with epilepsy are medically refractory, and most are treated with polytherapies (Kwan and Brodie, 2000). Optimisation of polytherapy and rational combination of ASMs remains a controversial issue. Higher drug load is usually a reflection of a more severe epilepsy, but polytherapy, or too high a dose of ASMs in monotherapies, can do more harm in terms of side effects (SE) than good in terms of seizure control (Perucca and Kwan, 2005). The cognitive effect of ASMs increased when drugs are combined, as shown on neuropsychological testing (Rösche et al., 2011; Phabphal and Kanjanasatien, 2011; Witt and Helmstaedter, 2013; Witt et al., 2015), but these studies usually include ASMs with known severe or moderate cognitive SEs.

Cognitive difficulties may be more disabling than seizures, significantly affecting quality of life (Baxendale and Thompson, 2016). ASMs are, often chosen not because of their perceived efficacy but because of their cognitive SE profile. Certain ASMs, such as topiramate, specifically have a negative effect on verbal fluency and working memory (Ojemann et al., 2001; Mula and Trimble, 2009). Most new-generation ASMs may exert mild or moderate cognitive SEs. It remains unclear whether and how these traditionally viewed cognitively “benign” ASMs affect cognition when given in polytherapy.

Language functional magnetic resonance imaging (fMRI) elicits consistent and reproducible patterns of activation and deactivation in response to language tasks and is in clinical use for pre-operative language lateralisation in epilepsy (Woermann et al., 2003; Duncan et al., 2016). Previous language fMRI studies examining the effects of different ASMs on cognitive activation and deactivation patterns showed (1) decreased activation in task-positive regions, i.e., dominant inferior and middle frontal gyri (IFG and MFG), and (2) failure to deactivate task-negative regions, including default mode network (DMN) areas (Jokeit et al., 2001; Szaflarski et al., 2010; Yasuda et al., 2013; Wandschneider et al., 2017). This was observed even in people taking ASMs with moderate cognitive SEs, such as levetiracetam (LEV), carbamazepine (CBZ), and lamotrigine (LTG) when compared with healthy controls (Xiao et al., 2018). To date, no study has addressed the effects of polytherapy and of dosage on cognitive activation patterns.

Here, we compare differences in fMRI activation patterns in people with focal epilepsy, matched for treatment propensity, to address the effects of taking ASM.

(i) Either with moderate [LEV, LTG, CBZ, oxcarbazepine (OXC), eslicarbazepine (ESLI), lacosamide (LCM), and valproic acid (VPA)] or with severe cognitive SEs (topiramate and zonisamide).
(ii) With moderate SEs as mono- or polytherapies.
(iii) With moderate SEs in different dosages.

We hypothesise that,

(i) People taking ASM with moderate adverse cognitive effects will show stronger fMRI activation on cognitive tasks in task-positive areas than those taking ASM with severe adverse cognitive effects.
(ii) Each additional ASM given in polytherapy will have a measurable effect on cognitive activation patterns.
(iii) With increasing drug-load there will be reduced activation on cognitive fMRI in task-positive areas and failure to deactivate in task-negative areas, irrespective of whether ASMs are given in high-dose monotherapy or low-dose polytherapy.

## Materials and Methods

### Participants

We analysed routinely acquired fMRI data of adults with refractory focal epilepsy who had clinical language fMRI scans as part of their pre-surgical evaluation at the National Hospital for Neurology and Neurosurgery (NHNN), London, United Kingdom, between January 2010 and January 2017. Presumed lateralisation and localisation of epilepsy was based on the clinical workup by experienced clinicians, which included semiological, electrophysiological, and neuroimaging data, along with ictal EEGs during video EEG telemetry or ambulatory long-term EEG monitoring. Ambiguous or unclear lateralisation or localisation was classified as undetermined.

Inclusion/exclusion criteria: Participants had to be literate and proficient in English, and be able to understand the simple task instructions. We excluded people with excessive motion or failure to perform verbal fluency tasks. We excluded people with brain lesions other than hippocampal sclerosis, to control for the potential remote effect of lesions on neuronal networks. People with severe cognitive deficits (IQ < 70), as reported by two experienced neuropsychologists without awareness of fMRI activation patterns, were excluded from further analysis. People were excluded if they were under treatment of psychiatric medications but not excluded if they have history of depression or anxiety. People were excluded from further analysis if they had undergone changes in ASM in the year before the scan date.

Based on our previous cross-sectional fMRI studies (Vollmar et al., 2011; Yasuda et al., 2013; Wandschneider et al., 2014; Wandschneider et al., 2017; Xiao et al., 2018) and neuropsychological studies focussing on the SE profiles of ASMs (Ojemann et al., 2001; Mula and Trimble, 2009) people were divided into two groups according to the number and types of ASM taken at the time of fMRI scanning:

(1) Marked cognitive SE (“severe”): topiramate and zonisamide (Mula and Trimble, 2009).
(2) Cognitive SE (“moderate”), which included LEV, LTG, CBZ, OXC, ESLI, LCM, and VPA.

Routine assessments of language function were available for a subset of subjects, but did not necessarily occur at the same time of scanning, with some having more than one assessment on different dates. We selected the assessment results closest to the scan date. These tests included category fluency test, letter fluency test and Graded Naming Test for expressive language function; National Adult Reading Test (NART) to provide an estimate of premorbid intellectual intelligence quotient (Bird et al., 2004).

The Joint Ethics Committee of the NHNN and University College London Institute of Neurology approved the study. The Committee classified this work as an evaluation of clinical services, and therefore individual consent was not required.

### Magnetic Resonance Imaging Data Acquisition, Functional Magnetic Resonance Imaging Paradigm, and Pre-processing

Gradient echo-planar images providing blood oxygen level-dependent (BOLD) contrast was acquired on a 3T Excite HDx scanner (General Electric), using a standard 8-channel receive coil. A scanner upgrade took place in 2013, but the scanning protocol remained unchanged. Each volume comprised 50 contiguous oblique axial slices, ensuring full brain coverage, with 2.5-mm slice thickness, 64 × 64 matrix, and 24-cm field of view, providing an in-plane voxel size of 3.75 × 3.75 mm. Echo time was 25 milliseconds, and repetition time was 2.5 s. Subjects performed a covert verbal fluency task lasting for 5 min. During the paradigm, 30-s blocks of task were alternated with 30-s blocks of crosshair fixation as a control condition. Participants were instructed to covertly generate words starting with a visually presented letter (A, D, E, S, and W). On our scanner, we can review a real-time activation map to assess compliance with the task. If there is no obvious activation map, the radiographer would repeat the scan.

Functional magnetic resonance imaging data were pre-processed with Statistical Parametric Mapping 12 (SPM12), version 6906^1^, including realignment, spatial normalisation to a scanner- and acquisition-specific template in the Montreal Neurological Institute (MNI) space 152, resampling to isotropic 3 × 3 × 3 voxels, and spatial smoothing (Gaussian kernel, 8 mm full width at half maximum). At the individual-subject level, the task was modelled by convolving the vector of block onsets with a canonical hemodynamic response function to create regressors of interest, and 6 motion parameters were included as confounds. Contrast images for each participant were created for task-relevant activation and deactivation associated with generating words.

### Drug Dosage Normalisation

To assess the effect of high or low doses of ASMs when given in either monotherapy or polytherapy, we normalised the drug load for each drug according to the British National Formulary^2^, using a scale from 1 (starting dose) to 10 (maximal recommended dose). Up to two points were added to this scale if a dose higher than the maximum recommended dose was prescribed (11 = 2nd highest dose prescribed across the whole sample; 12 = highest dose prescribed overall). Table 1 provides the details of the drug load score for each ASM.

**TABLE 1:** ASM drug load score system.

Medication load score	CBZ	ESL	LCM	LEV	LTG	OXC	VPA
1	200 mg	400 mg	100 mg	500 mg	50 mg	300 mg	400 mg
2	400 mg	500 mg	150 mg	750 mg	100 mg	450 mg	600 mg
3	600 mg	600 mg	200 mg	1,000 mg	150 mg	600 mg	800 mg
4	800 mg	7000 mg	250 mg	1,250 mg	200 mg	750 mg	1,000 mg
5	1,000 mg	800 mg	300 mg	1,500 mg	250 mg	900 mg	1,200 mg
6	1,200 mg	900 mg	350 mg	1,750 mg	300 mg	1,200 mg	1,400 mg
7	1,400 mg	1,000 mg	400 mg	2,000 mg	350 mg	1,500 mg	1,600 mg
8	1,600 mg	1,200 mg	450 mg	2,250 mg	400 mg	1,800 mg	1,800 mg
9	1,800 mg	1,400 mg	500 mg	2,500 mg	450 mg	2,100 mg	2,000 mg
10	2,000 mg	1,600 mg	600 mg	3,000 mg	500 mg	2,400 mg	2,500 mg
11	2,600 mg	1,800 mg	700 mg	3,500 mg	650 mg	2,700 mg	3,000 mg
12	3,200 mg	2,000 mg	800 mg	4,000 mg	800 mg	3,000 mg	3,500 mg

*The medication load is normalised for each ASM according to British National Formulary (BNF) guidelines (https://bnf.nice.org.uk/drug/). CBZ, carbamazepine; ESLI, eslicarbazepine; LCM, lacosamide; LEV, levetiracetam, LTG, lamotrigine TPM, topiramate; OXC, oxcarbazepine VPA, valproic acid; ZNS, zonisamide.*

### Statistical Analysis

To minimise treatment bias, we used propensity score matching (PSM) in SPSS 22 (IBM Corp.) and balanced treatment groups for age, sex, epilepsy duration, handedness (assessed via the Edinburgh Handedness Inventory), history of generalised seizures, history of febrile seizures, hippocampal sclerosis, lateralisation and localisation of epilepsy and seizure frequency. Age, history of generalised and febrile seizures and hippocampal sclerosis are binary. Handedness, lateralisation and localisation of epilepsy and seizure frequency are in category. Binary and category details are in Tables 1, 2. Age and epilepsy duration are continuous. All subjects were anonymised before PSM and options for priority are given to exact matches, and maximum randomness was selected during the procedure. We started with the dual-therapy group with “severe” ASM and looked for matched subjects taking dual-therapy of “moderate” ASMs. In analysis of mono vs. polytherapy with “moderate” ASMs, we looked for matched mono- and dual-therapy groups with the triple-therapy group. Each matched group was randomly and exactly matched to the target group.

**TABLE 2:** Demographic and clinical features between ASMs with moderate and severe side effects.

	ASMs with severe side effects (*n* = 119)	ASMs with moderate side effects (*n* = 119)	*P-*value
Sex, F/M	56/63	56/63	1.000
Age at the scan (SD), years	34.5 (10.7)	35.2 (11.5)	0.623
Epilepsy duration at the scan (SD), years	14.7 (10.2)	15.1 (11.9)	0.748
Handedness, Right/Left/Ambidextrous	101/16/2	103/13/3	0.767
Localisation of epilepsy, *n*, (%)			0.273
Temporal	70 (58.8)	76 (63.9)	
Frontal	28 (23.5)	30 (25.2)	
Parietal	7 (5.9)	8 (6.7)	
Occipital	1 (0.8)	1 (0.8)	
Undetermined	13 (10.9)	4 (3.4)	
Lateralisation of epilepsy, *n*, (%)			0.658
Left	61 (51.3)	62 (52.1)	
Right	44 (37.0)	48 (40.3)	
Bilateral	6 (5.0)	5 (4.2)	
Undetermined	8 (6.7)	4 (3.4)	
History of febrile seizures, *n*, (%)	15 (12.6)	13 (10.9)	0.841
Hippocampal sclerosis, *n*, (%)	28 (23.5)	33 (27.7)	0.553
Seizure frequency, *n*, (%)			0.619
Less than once a month	8 (6.7)	9 (7.6)	
Monthly to weekly	32 (26.9)	31 (26.1)	
Weekly to daily	50 (42.0)	49 (41.2)	
Daily seizures	29 (24.4)	30 (25.2)	
History of GTCS, *n*, (%)	82 (68.9)	77 (64.7)	0.582
Clinical language assessments			
Letter fluency test, mean (SD)	*n* = 71, 11.86 (7.17)	*n* = 68, 13.59 (5.22)	0.108
Category fluency test, mean (SD)	*n* = 73, 16.37 (6.39)	*n* = 69, 17.39 (7.62)	0.191
NART, mean (SD)	*n* = 49, 95.33 (12.37)	*n* = 52, 98.53 (11.05)	0.237
Graded naming test, mean (SD)	*n* = 65, 15.60 (6.19)	*n* = 62, 15.57 (5.82)	0.949
Scanner, original/upgrade	43/76	37/82	0.496

*ASM, anti-seizure medication; NART, National Adult Reading Test; GTCS, generalised tonic clonic seizures; SD, standard deviation.*

For the fMRI group-level analysis, we first compared people taking “severe” or “moderate” ASMs. We further investigated the effect of the number of ASMs on cognitive patterns by comparing people on monotherapy, dual therapy and triple therapy of “moderate” ASMs. We entered activation contrasts for each individual into a full factorial design with group as a factor [(“moderate” and “severe”), (“mono-,” “dual-,” and “triple-” therapy)], and performed the following analyses: (1) a *t*-test comparing “moderate” versus “severe” in dual-therapy; and (2) a one-way ANOVA comparing groups taking “moderate” ASMs only in mono-, dual-, or triple-therapy. We anatomically objectified peak activations from group comparisons with coordinates in the MNI template. The significance threshold was set at *P* < 0.005 uncorrected, 20 voxels extend threshold for group comparisons.

Across people taking moderate ASMs, multiple regression models were implemented within SPM to assess correlations between the clinical variables including (age, duration, sex, handedness, atypical hemispheric dominance, history of generalised seizures, hippocampal sclerosis, and seizure frequency) and activation and deactivation patterns during verbal fluency. Seizure frequency was classified as yearly-monthly/monthly-weekly/weekly-daily/daily (1–4) and a history GTCS was classified (yes/no) and entered in a full factorial multiple regression model to test for the effect of seizure severity. To investigate the effect of drug dosage of individual moderate ASMs on fMRI activation, we ran separate multiple regression analyses, one per moderate ASM, in which we correlated each subject’s whole-brain functional MRI patterns with drug dosage of that ASM. To examine the effect of the combined drug load of ASMs with moderate SEs (*n* = 180), individual drug load scores were correlated against each subject’s whole-brain functional MRI patterns in one multiple regression model in SPM. For correlation analyses, we hypothesised that a higher drug load would relate to stronger suppression of the fMRI task signal, echoing prior work in neuropsychology which showed a detrimental influence of high drug load on cognitive test scores (Witt and Helmstaedter, 2013; Witt et al., 2015). Specifically, we hypothesised that specific regions of interest (ROIs) that undergo task-related activation/deactivation would exhibit negative/positive correlations with drug load, respectively. In SPM, correlational analyses are one-tailed by default; indeed, we reported them exactly as provided by the software (one-tailed), in view of the above a priori hypothesis. An exploratory statistical threshold was set at *p* < 0.005 uncorrected with a 10-voxel minimum cluster size extent threshold, for positive and negative correlations separately (Lieberman and Cunningham, 2009; Trimmel et al., 2018; Caciagli et al., 2019). In case an association did not reach statistical significance at conventional thresholds that account for correction for multiple comparisons (e.g., *p* < 0.05, familywise error corrected), we explicitly considered the former as providing “exploratory evidence.”

ANOVA was used for continuous and normally distributed data and Kruskal–Wallis or Mann–Whitney were used for non-normally distributed data. Continuous variables are displayed as mean (SD) for normally distributed data Calculations were done in SPSS 22.0 (IBM Corp.).

### Data Availability

The data supporting the findings of this study are available from the corresponding author upon reasonable request. The raw data are not publicly available because of ethical restrictions.

## Results

In total, 960 people with focal epilepsy underwent language fMRI during the study period. We excluded people with insufficient fMRI activation due to excessive motion (*n* = 166) or failure to perform verbal fluency task (*n* = 103), large brain lesions (*n* = 121), severe cognitive deficits or with active treatment of psychiatric medications (*n* = 83), as well as drug changes within the year preceding the scan (*n* = 15). Of 472 people eligible for further analysis, 335 were taking ASMs with moderate SE (85 on moderate ASM monotherapy, 190 on two moderate ASMs, 60 on three or more moderate ASMs) and 137 with severe cognitive SE (8 on one severe ASM in monotherapy; 119 on two ASMs, either one moderate and one severe ASM, or two severe ASMs in dual-therapy; 10 on three or more ASMs, of which at least one was a severe ASM). Comparing ASMs with moderate and severe cognitive SE, we focused on the dual therapy group (119 vs. 119), due to small number on mono- or triple-therapy with severe cognitive SEs. Comparing the effect of mono- versus poly-therapy among those people taking ASMs with moderate cognitive SE, we included 60 individuals each for mono-, dual-, or triple-therapy groups (taking moderate ASMs only) after PSM analysis. There were 38 people taking dual-therapy had been included in the previous analysis for moderate vs. severe ASMs. In total, 380 people were included in the final analysis. Pre-PSM demographic and clinical characteristics are provided in Supplementary Tables 1, 2.

### Comparison of Anti-seizure Medications With “Moderate” or “Severe” Cognitive Side Effect

**FIGURE 1:**
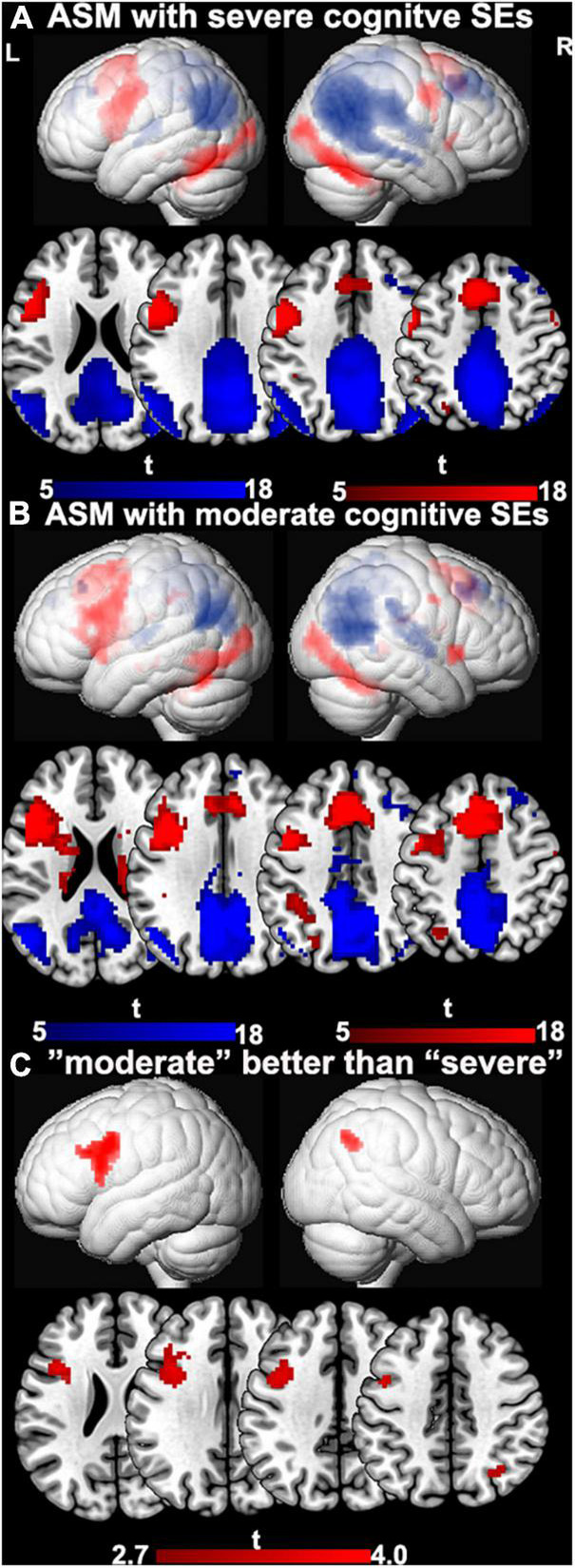
The difference between ASMs with “moderate” and “severe” cognitive side-effects. **(A)** One-sample *t-*tests of functional magnetic resonance imaging activation and deactivation maps for the therapy groups with ASMs with “severe” cognitive side-effects are demonstrated on a surface-rendered brain template and subcortical changes are demonstrated superimposed on MNI 152 template with a bar chart indicating *t* score. Task-relevant activations (red) include bilateral inferior and middle frontal gyrus (left > right), bilateral supplementary motor area, the left dorsolateral parietal region, and bilateral inferior occipital lobes. **(B)** One-sample *t-*tests of functional magnetic resonance imaging activation and deactivation maps for the therapy groups with ASMs with “moderate” cognitive side-effects are demonstrated on a surface-rendered brain template and subcortical changes are demonstrated superimposed on MNI 152 template with a bar chart indicating *t* score. Task-relevant activations (red) include bilateral inferior and middle frontal gyrus (left > right), bilateral supplementary motor area, the left dorsolateral parietal region, and bilateral inferior occipital lobes. Areas of task-related deactivations (blue) include the bilateral precuneus, posterior cingulate, angular gyrus, and medial prefrontal and lateral temporal cortex. *P* < 0.05, FWE-corrected. **(C)** Significant group differences between ASMs with “moderate” and “severe” cognitive side-effects are demonstrated on a surface-rendered brain template and subcortical changes are demonstrated superimposed on MNI 152 template with a bar chart indicating *t* score. People on ASM with moderate SE profiles show greater activation in left frontal verbal fluency networks and greater deactivations in right inferior parietal lobe than people with severe SE profiles. *P* < 0.005, uncorrected, extent threshold 20 voxels. ASM, anti-seizure medication; L, left; R, right; SE, side effect.

Demographic and clinical characteristics are provided in Table 2. One-sample *t-*tests of task-relevant activations and deactivations for each group are shown in Figure 1A. In people taking “moderate” ASMs, we observed higher activation of the IFG, MFG, and lower deactivation of the right inferior parietal lobule than in people taking “severe” ASMs, (*P* < 0.005 uncorrected, 20 voxels) (Figure 1B, statistics in Table 2).

### Effect of Mono-, Dual-, or Triple Therapy With Anti-seizure Medications With “Moderate” Cognitive Side Effect on Cognitive Activation Patterns

Table 3 provides demographic and clinical details. There were no significant differences between the three groups of people taking either monotherapy or combinations of two or three ASMs with moderate cognitive SEs. No clinical factors were significantly associated with functional activation or deactivation maps. Different dosages of ASMs with moderate cognitive SE did not affect task-related activation and deactivation patterns.

**TABLE 3:** Demographic and clinical features of people taking ASMs with moderate cognitive effects in mono/dual/triple therapy.

	Monotherapy (*n* = 60)	Dual therapy (*n* = 60)	Triple therapy (*n* = 60)	*P-*value
Sex, F/M	28/32	26/34	23/37	0.622
Age at the scan (SD), years	34.0 (9.3)	32.5 (9.5)	33.3 (10.4)	0.686
Epilepsy duration at the scan (SD), years	14.5 (11.1)	13.8 (10.8)	14.6 (9.7)	0.910
Handedness, Right/Left/Ambidextrous	54/6	56/4	54/6	0.760
Localisation of epilepsy, *n*, (%)				0.774
Temporal	43 (71.7)	41 (68.3)	43 (71.7)	
Frontal	11 (18.3)	15 (25.0)	14 (23.3)	
Parietal	6 (10)	4 (6.7)	3 (5.0)	
Lateralisation of epilepsy, *n*, (%)				0.672
Left	31 (51.7)	33 (55.0)	30 (50.0)	
Right	26 (43.3)	19 (31.6)	23 (38.3)	
Bilateral	2 (3.3)	7 (11.7)	6 (10.0)	
Undetermined	1 (1.7)	1 (1.7)	1 (1.7)	
History of febrile seizures, *n*, (%)	6 (10.0)	7 (11.7)	5 (8.3)	0.831
Hippocampal sclerosis, *n*, (%)	13 (21.7)	22 (31.9)	15 (25.0)	0.156
Seizure frequency, *n*, (%)				0.843
Less than once a month	7 (11.7)	6 (10.0)	4 (6.7)	
Monthly to weekly	19 (31.7)	19 (31.7)	15 (25.0)	
Weekly to daily	20 (33.3)	19 (31.7)	25 (41.7)	
Daily seizures	14 (23.3)	16 (26.6)	16 (26.6)	
History of GTCS, *n*, (%)	39 (65.0)	45 (75.0)	39 (65.0)	0.397
Scanner, original/upgrade, *n*	21/39	17/43	16/44	0.577
Clinical language assessments				
Letter fluency test, mean (SD)	*n* = 40, 14.13 (5.48)	*n* = 36, 14.17 (5.69)	*n* = 42, 12.90 (4.76)	0.480
Category fluency test, mean (SD)	*n* = 40, 18.55 (6.39)	*n* = 36, 18.39 (5.06)	*n* = 42, 18.00 (5.84)	0.908
NART, mean (SD)	*n* = 40, 99.33 (14.08)	*n* = 36, 99.44 (9.97)	*n* = 42, 94.88 (12.82)	0.177
Graded naming test, mean (SD)	*n* = 40, 15.45 (6.35)	*n* = 36, 15.47 (5.34)	*n* = 42, 14.64 (5.86)	0.770
Drug load score, median (range)	6.5 (1.00−12.00)	13.00 (2.50–24.00)	18.5 (7.00–35.00)	<0.001

*ASM, anti-seizure medication; NART, National Adult Reading Test; GTCS, generalised tonic clonic seizures; SD, standard deviation.*

**FIGURE 2:**
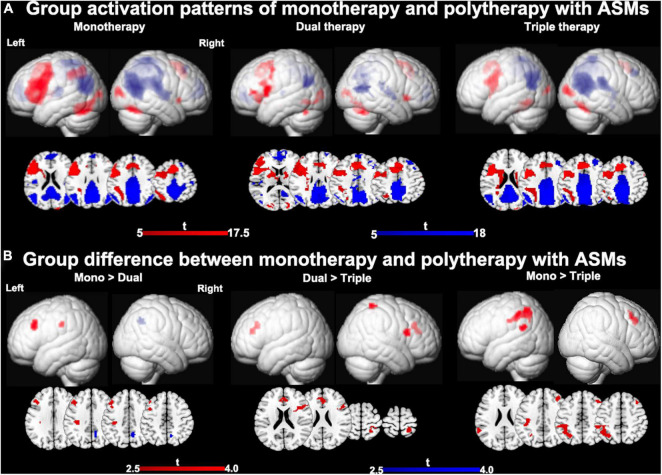
Main effects of number of ASMs with moderate cognitive side effects on cognitive activation patterns. **(A)** One-sample *t-*tests of functional magnetic resonance imaging activation and deactivation maps for the three different therapy groups are demonstrated on a surface-rendered brain template and subcortical changes are demonstrated superimposed on MNI 152 template with a bar chart indicating *t* score. Task-relevant activations (red) include bilateral inferior and middle frontal gyrus (left > right), bilateral supplementary motor area, the left dorsolateral parietal region, and bilateral inferior occipital lobes. Areas of task-related deactivations (blue) include the bilateral precuneus, posterior cingulate, angular gyrus, and medial prefrontal and lateral temporal cortex. *P* < 0.05, FWE-corrected. **(B)** Significant group differences between monotherapy and polytherapy are demonstrated on a surface-rendered brain template and subcortical changes are demonstrated superimposed on MNI 152 template with a bar chart indicating *t* score. People on monotherapy show greater activation in frontal cognitive networks and deactivation in task-negative networks than people with 2 ASMs. People on monotherapy or on two ASMs show greater frontal and parietal activations than people on 3 ASMs. *P* < 0.005 uncorrected, extent threshold 20 voxels. ASM, anti-seizure medication.

One-sample *t-*tests of task-relevant activations and deactivations for each group are demonstrated in Figures 2A,B. As for group comparisons (Figure 1C and Table 4), people on monotherapy showed greater activation of frontal areas and greater deactivation of task-negative areas than people with 2 ASMs (*P* < 0.005 uncorrected, 20 voxels). Those on monotherapy or on two ASMs show greater frontal or parietal activation and lower parietal deactivation than people on 3 ASMs (*P* < 0.005 uncorrected, 20 voxels).

**TABLE 4:** Anatomic description and peak activations of resultant areas from group comparisons.

Regions	MNI coordinates (*x*, *y*, *z*)	*Z* score	*P-*value
**Moderate > severe side effects**			
Increased activation: Left inferior frontal gyrus	−39, 11, 22	3.62	<0.001
Decreased deactivation: Right Inferior parietal lobule	33, −58, 37	2.79	0.003
**Mono > Dual therapy**			
Increased activation: Left middle frontal gyrus	−42, 29, 34	3.20	0.001
Increased activation: Left inferior parietal lobule	−36, −25, 37	3.17	0.001
**Mono < Dual Therapy**			
Decreased deactivation: Right precuneus	9, −58, 43	3.01	0.001
**Dual > Triple Therapy**			
Increased activation: Anterior cingulate cortex	−9, 41, 19	2.93	0.002
Increased activation: Right superior parietal lobule	24, −49,67	3.40	<0.001
Increased activation: Right inferior frontal gyrus	45, 14, 19	3.37	<0.001
**Mono > Triple therapy**			
Increased activation: Left inferior parietal lobule	−48, −55, 55	3.16	0.001
Increased activation: Left inferior frontal gyrus	−42, 29, 43	3.17	0.001
Increased activation: Left supramarginal gyrus	−63, −49, 22	3.04	0.001
Increased activation: Right middle frontal gyrus	33, 32, 37	2.80	0.002

*Coordinates are given in MNI space. ASM, anti-seizure medication; LEV, levetiracetam; LTG, lamotrigine; MNI, Montreal Neurological Institute. The results reported here are uncorrected.*

### Effects of Drug Load Score on Mono-, Dual-, or Triple Therapy of Anti-seizure Medications With “Moderate” Cognitive Side Effect

There is a significant difference across the three groups at drug load scores (*F* = 82.05, *P* < 0.001) (Table 3). The post-hoc analysis showed that monotherapy’s drug load score is lower than dual or triple therapy with ASMs with “moderate” cognitive SE. The drug load score of dual-therapy is lower than triple-therapy (all *p* < 0.001, Bonferroni-corrected) (Supplementary Figure 1).

**FIGURE 3:**
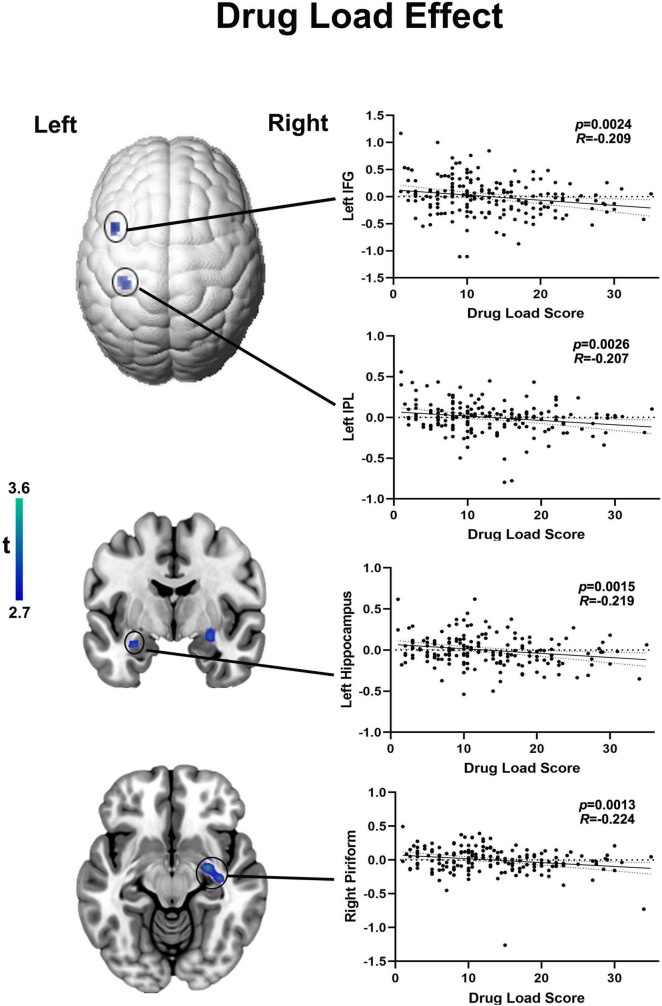
Functional activation in language areas and piriform cortex correlates with drug load of ASMs with moderate cognitive side effects. Cortical changes are demonstrated on a surface-rendered brain template and subcortical changes are demonstrated superimposed on MNI 152 template with a bar chart indicating *t* score. Among those on moderate ASMs, the drug load score is negatively correlated with functional activation in right piriform cortex left hippocampus, higher drug dosage scores were associated with lower activation of the inferior frontal gyrus (IFG) and left inferior parietal lobule (IPL) at a threshold of *P* < 0.005 uncorrected, 10 voxels.

Among those on ASMs with moderate SE (*n* = 180), no regions survived positively in correlation but there are four region of interests were detected negatively correlated with drug load score at the exploratory threshold of *p* < 0.005 uncorrected, 10 voxel. Higher drug dosage scores were associated with lower activation of: (i) the right piriform cortex (*R* = −0.224, *p* = 0.0013, uncorrected, 39 voxels; peak MNI coordinates: 27, −7, 1), (ii) the left hippocampus (*R* = −0.219, *p* = 0.0015, uncorrected, 20 voxels; peak MNI coordinates: −30, −4, −20), (iii) the left inferior frontal gyrus (IFG) (*R* = −0. 209, *p* = 0.0024 uncorrected, 12 voxels; peak MNI coordinates: −45, 11, 43) and (iv) the left inferior parietal lobule (IPL) (*R* = −0.207, *p* = 0.0026 uncorrected, 11 voxels; peak MNI coordinates: −35, −34, 43) (Figure 3). As none of the above correlations reached statistical significance after familywise error correction for multiple comparison, the latter evidence needs to be interpreted as “exploratory.” The 22 outliers in this analysis were removed and the analysis was redone, generating similar results (Supplementary Figure 2).

## Discussion

We show that ASMs are associated with altered cognitive activation patterns during verbal fluency, most marked for drugs known having more severe cognitive SE profiles. ASMs considered to have moderate cognitive SEs had an effect on fMRI activation patterns when given in polytherapy. A large dose of mono- or poly-therapy also adds to the cognitive effect of people taking ASMs with moderate cognitive SEs.

After excluding ASMs suspected to cause clinically relevant cognitive impairment and focusing only on drugs with supposedly moderate cognitive SEs, we found that the number of ASMs negatively affected functional activation patterns, irrespective of the specific ASMs taken. When given in polytherapy, ASMs considered to be “moderate” led to reduced activation of frontal regions relevant for language function and decreased deactivation of task-negative default mode regions. This is similar to effects previously described for ASMs associated with severe SE, like topiramate and zonisamide (Wandschneider et al., 2017). Comparing people taking two ASMs with moderate cognitive SEs to people treated with two ASMs including either topiramate or zonisamide, we observed reduced activation in language areas. This corroborates our hypothesis that, for the same number of concurrent ASMs, people treated with drugs with severe cognitive SEs may show more altered activation patterns during cognitive task than those on ASM with moderate cognitive SEs.

While we found differences in activation patterns across three groups on ASMs with moderate SEs during verbal fluency fMRI scanning, there were no significant differences in clinical language measures. People with epilepsy on ASMs with either severe or moderate cognitive SEs show more cognitive decline than healthy controls. In our previous studies (Yasuda et al., 2013; Wandschneider et al., 2014; Xiao et al., 2018), when comparing across people with epilepsy, there was little difference in clinical assessments despite the significant difference in activation and deactivation patterns in verbal fluency fMRI. There are a few possible interpretations due to the limitation of the cognitive data. Although the difference in neuropsychological tests is not significantly important, the trend in all the are decreasing with the increase of drug load and number of medications. We did sub-analysis including only those who had both cognitive data and fMRI and showed similar significant but less difference (Supplementary Figure 7). Ideally, out of scanner cognitive tests should have be done on the same day of the scan but few participants had them both the same day. A limited number of participants underwent the tests within the 6 months’ of the scanning. Thus, the neuropsychological tests here are a background descriptor. Another possible explanation could be that the changes may be a BOLD-related phenomenon such as changed or increased resting cerebral blood flow. Alternatively, our findings are not confounded by differences in performance but directly related to the drugs’ effects, which supports our hypothesis that drug load has an effect on cognitive function. The difference in this study between the different numbers of “moderate” ASMs is relatively subtle unlike the ASMs with pronounced cognitive side effects such as topiramate and zonisamide, specifically affecting clinical expressive language ability. Hence, the affected regions may not be as similar and specific as ASMs with serious cognitive SEs. The future cognitive fMRI studies along with the same day’s cognitive tests investigating same domains will better disentangle this question. We propose the use of verbal fluency fMRI tasks as a robust tool, in addition to neuropsychological batteries, to assess more comprehensively the cognitive effect of ASMs. While difficulties hinder the immediate implementation of fMRI as a standard tool at the individual-level to quantifying limits of normal and abnormal activations at the single-subject level, we note that efforts to deliver individual-level fMRI biomarkers are ongoing and promising (Gordon et al., 2017).

Drug load was defined as the total amount of drug exposure for a given treatment indication (Deckers et al., 1997; Witt et al., 2020). WHO attempted to quantify “drug load” in epilepsy as the prescribed daily dose; using it a marker, side effects were more linked with overall drug load than with the drug number (Deckers et al., 1997). In our study, we used the local gold standard (BNF) to normalise each ASM’s drug dosages and obtain drug load scores. After accounting for the effects of drug dosages, increased drug load score correlated with reduced functional activation of left hippocampus left IPL and left IFG during verbal fluency. Activation of these brain areas plays a crucial role in language fMRI tasks (Glikmann-Johnston et al., 2015; Wandschneider et al., 2017; Xiao et al., 2018; Caciagli et al., 2020). People with damage to, or after removal of the left hippocampus typically present impaired semantic fluency, i.e., the left hippocampus is not necessary for this task as the person can still generate words. Whilst fMRI is different from WADA testing, it is still able to show the disruptive effects of lesions or failed segregation of certain parts of networks on cognitive task (Fahoum et al., 2012). This suggests suppression of several language-related cognitive domains with increased drug load. As the individual drug load increases with the number of ASMs, our correlation analysis indicates that a high dose of one ASM has similar effects on cognitive activation patterns than a combination of two ASMs, both given in low doses.

A possible explanation for our observation of fMRI activity altered by polytherapy in epileptogenic and cognitive networks is that multiple ASMs may strengthen the brain’s intrinsic inhibitory countermeasures to suppress seizures (Thomas et al., 2006; French and Faught, 2009). In previous neuropsychological studies, simply counting the number of ASMs may be sufficient as a rough estimate of the risk of cognitive SEs (Witt and Helmstaedter, 2013; Witt et al., 2015). All these findings were based on the inclusion of ASMs with severe and moderate cognitive SEs without correction of influences of ASMs with severe cognitive SEs. We demonstrate the probable neural correlates cognitive effect of ASMs commonly considered cognitively “safe” to use.

Our finding of increased drug load associated with reduced BOLD response in the piriform cortex is similar to previous EEG-fMRI studies (Laufs et al., 2011; Fahoum et al., 2012; Flanagan et al., 2013). A recent proof of concept study on the piriform cortex’s role for seizure modulation in TLE reported that the chance of seizure freedom after anterior temporal lobe resection increased 16-fold if at least 50% of the piriform cortex had been resected (Galovic et al., 2019). Akin to the original experimental study (Piredda and Gale, 1985), we speculate that ASM may exert their anti-seizure effects by reducing the piriform cortex’s activation, which likely represents a common node of focal epilepsy networks and contributes to the dissemination and amplification of epileptic discharges arising from mesial temporal structures. Within the context of language-related fMRI activations, reports of piriform cortex involvement in language fMRI studies are relatively scarce. In our study, the group effects of verbal fluency fMRI of people on monotherapy and polytherapies of ASMs with moderate SEs show that the right piriform is involved within the activation patterns during the task (Supplementary Figures 3–5).

### Strength and Limitations

Our study’s strengths are the inclusion of a large, diverse, and clinically representative tertiary centre cohort of people with refractory focal epilepsy and the use of a robust cognitive fMRI already established in clinical routine (Black et al., 2017; Szaflarski et al., 2017). All participants had focal epilepsy, but different epilepsy syndromes and seizure frequency ranges were included to obtain a representative cohort. We utilised PSM to minimise treatment selection bias, an approach that mimics some aspects of a randomised controlled trial (Austin, 2008). Additionally, demographic and clinical variables were included as regressors of no interest in the fMRI analysis model. Not all potential unmeasured confounders could be accounted for by PSM. All people had refractory focal epilepsy syndromes and had tried various ASMs with the dosage of each ASM titrated to the best possible effect level. Depending on the minimum and maximum dosage of each ASM, we created a medication load score for each ASM to normalise the drug load at an individual level irrespective of the number of the ASMs taken.

Our study is limited by its retrospective nature. The covertly conducted fMRI paradigm is another limitation, but we excluded the people with the lack of activations of bilateral MFG or IFG. The interpretation of neuroimaging studies in psychologically impaired people depends on intact task performance and detailed task analysis. Only when these criteria are met studies can be used to identify dysfunction and compensation. Underactivity on fMRI is only suggestive of dysfunction, or overactivity suggestive of compensation, when the different low and high drug-load groups make comparable responses. In addition, to better help present the “abnormal” activation and deactivation map we have added a comparison of VF fMRI between healthy controls (*n* = 62) and patients with “severe” (*n* = 60) and patients with “moderate” (*n* = 60) ASMs group to define “abnormal” activation and deactivation map in the Supplementary Figure 6.

The statistical threshold (*P* < 0.005 with 20-voxel threshold extent) used for group-level models and correlation analyses was uncorrected, but nonetheless enables an exploratory view of the differences between ASM treatment groups. We note that we used a corrected statistical threshold (*P* < 0.05, FWE-corrected across the whole brain voxel-wise) in our individual fMRI preprocessing, to make sure that group analyses would only involve activation maps with robust statistics. We submit that a statistical threshold of *P* < 0.05, FWE-corrected voxel-wise across the whole brain is stricter than standards for individual-level exclusion in prior work. We found no difference of ASM number and drug load score in the mono-, dual-, and triple-therapy groups of patients included and excluded from this study (Supplementary Table 1). Hence, the lack of activation in these patients is unlikely due to the effects of polytherapy and drug load, but, we speculate, due to movement and inability to perform the tasks owing to low concentration, anxiety or other undetermined reasons.

Cognitive function is not only affected by ASMs but also by aetiology, particularly for lesions affecting eloquent areas, as well as by seizures and mood disturbances. We attempted to control for these effects by excluding participants with (1) lesions other than hippocampal sclerosis; (2) psychiatric disorders with ongoing treatment of psychiatric medications; (3) severe cognitive impairment (IQ < 70), who were not able to engage in the task.

The performance in NART reading tests is stable over a lifetime, but the performance in clinical verbal fluency tests is more dynamic and particularly susceptible to change over time due to factors including anxiety, depression, practice effects, fatigue, motivation and ASMs (Bird et al., 2004). To control for performance, we included the accessible clinical language measures closest to the scan date as a background descriptor. There was a discrepancy between the assessment dates and scan dates (29 out of 118 participants had an assessment within 6 months of the scan dates). For this reason, these assessments could not be used to validate the effects of ASMs on activation patterns externally through direct correlation analysis. It is challenging to rule out a potential influence of seizure activity on the cognitive effects described in our study. Future prospective longitudinal investigations with well-controlled seizure characteristics in ASM monotherapy may help further address the underlying mechanisms.

Personal prescribing choices could drive the ASM profile of our groups. A large number (>10) of consultant neurologists at our centre were involved in treatment decisions, minimising such bias. We summarised the ASM number and drug load score of the excluded candidates and found no difference between mono-, dual-, and triple therapy groups. We, however, acknowledge a referral bias, as only people with more severe epilepsy are usually referred for pre-surgical assessment.

### Conclusion

Our findings emphasise the concept that each additional drug matters concerning the cognitive effect of polytherapy (Witt et al., 2015), even if a given drug has moderate cognitive side effects. The selection of ASMs with moderate SEs in combined therapies may lessen the cognitive effect of polytherapy. Our *post hoc* correlation analysis suggests that apart from medication number, drug load is an important effect factor in polytherapy. Longitudinal studies with cognitive fMRI and neuropsychological data on the same day collection before and after the withdrawal of ASMs may better disentangle the effects of individual ASMs on seizure control and cognition. Our findings highlight the implications of adding ASMs and a high drug load, even if an individual ASM is considered to have a benign cognitive profile.

## Data Availability Statement

The original contributions presented in the study are included in the article/Supplementary Material, further inquiries can be directed to the corresponding author/s.

## Ethics Statement

The studies involving human participants were reviewed and approved by the Joint Ethics Committee of the NHNN and University College London Institute of Neurology. Written informed consent for participation was not required for this study in accordance with the national legislation and the institutional requirements.

## Author Contributions

FX and MK conceptualised and designed the study. FX, LC, SV, and AH carried out the acquisition and processing of imaging data. FX, LC, SV, BW, MG, LL, and DS contributed to imaging and statistical data analysis. SB, PT, and BJ collected and organised the clinical neuropsychological data. FX interpreted the data and drafted the manuscript. JD, JS, and MK supervised the data analysis, interpretation, and manuscript preparation. DZ and MK obtained funding. All authors contributed to data interpretation and manuscript preparation, had full access to all of the data, including statistical reports and tables in the study, and can take responsibility for the integrity of the data and data analysis accuracy, and approved the final version of the manuscript before submission.

## Conflict of Interest

JS has received personal fees from Eisai, UCB, GW Pharmaceuticals, Arvelle, and Zogenix, and research grants from UCB and GW Pharmaceuticals outside the submitted work. MK has received grants and honoraria from UCB Pharma, Desitin, Novartis, Eisai, GE, and Bial, outside the submitted work. MG has received honoraria from Bial Pharmaceutical and Nestlé Health Science outside the submitted work. The remaining authors declare that the research was conducted in the absence of any commercial or financial relationships that could be construed as a potential conflict of interest.

## Publisher’s Note

All claims expressed in this article are solely those of the authors and do not necessarily represent those of their affiliated organizations, or those of the publisher, the editors and the reviewers. Any product that may be evaluated in this article, or claim that may be made by its manufacturer, is not guaranteed or endorsed by the publisher.

## Footnotes

1
http://www.fil.ion.ucl.ac.uk/spm/

2
https://bnf.nice.org.uk/drug/
