# Supplementary material for: Effect of Anti-seizure Medications on Functional Anatomy of Language: A Perspective From Language Functional Magnetic Resonance Imaging
Source: Front Neurosci. 2022 Feb 24;15:787272. doi: 10.3389/fnins.2021.787272 (PMC8908426; doi:10.3389/fnins.2021.787272)
Supplement: Supplementary file 1 [file Data_Sheet_1.PDF]

Supplementary Contents

Supplementary Table 1. Demographic features between ASMs with moderate and severe side effects before PSM

Supplementary Table 2. Demographic and clinical features of patients taking ASMs with moderate cognitive effects in mono/dual/triple therapy before PSM

Supplementary Table 3. Drug load difference between excluded and included candidates on "moderate" ASMs

Supplementary Figure 1 The distribution of drug load across mono, dual and triple therapy groups with AMSs with moderate cognitive side effects

Supplementary Figure 2 Functional activation in language areas and piriform cortex correlates with drug load of ASMs with moderate cognitive side effects after removing the outliers

Supplementary Figure 3-5 The activation and deactivation patterns of people on anti-seizure medications (ASMs) with moderate cognitive SEs

Supplementary Figure 6 Comparison of VF fMRI between healthy controls (n=62) and patients with "severe" (n=60) and "moderate" (n=60) ASMs group

Supplementary Figure 7 Comparison of VF fMRI between those who had both fMRI and cognitive tests data

Supplementary Table 1 demographic features between ASMs with moderate and severe side effects before PSM

	ASMs with severe side effects (n= 119)	ASMs with moderate side effects (n= 190)	P value
Gender, F/M	56/63	84/106	0.355
Age at the scan (SD), y	34.3 (10.7)	34.6 (11.6)	0.420
Duration at the scan (SD), y	14.7 (10.2)	15.2 (11.4)	0.199
Handedness, R/L/A	75/9/1	163/22/5	0.693
Localization of epilepsy, n, (%)			0.008
Temporal	70 (58.8)	134 (70.5)	
Frontal	28 (23.5)	34 (17.8)	
Parietal	7 (5.9)	15 (7.9)	
Occipital	1 (0.9)	1 (0.3)	
Undetermined	13 (10.9)	4 (2.1)	
Lateralization of epilepsy, n, (%)			0.252
Left	61 (51.3)	83 (43.7)	
Right	44 (37.0)	86 (45.3)	
Bilateral	6(5.0)	14 (7.4)	
Undetermined	8(6.7)	7 (3.6)	
History of febrile seizures, n, (%)	15 (12.6)	19 (10.0)	0.297
Hippocampal sclerosis, n, (%)	25 (21.0)	39 (20.5)	0.313
Seizure frequency, n, (%)			0.253
Less than once a month, n, (%)	8 (6.7)	17 (8.9)	
Monthly to weekly	32 (26.9)	57 (30)	
Weekly to daily	50 (42.0)	71 (37.4)	
Daily seizures	29 (24.4)	45 (23.7)	
History of GTCS, n, (%)	81 (68.0)	127 (66.8)	0.402
Scanner, original/upgrade	45/79	81/109	0.236

Supplementary Table 2. Demographic and clinical features of patients taking ASMs with moderate cognitive effects in mono/dual/triple therapy before PSM

	Monotherapy (n= 85)	Dual therapy (n= 190)	Triple therapy (n= 60)	P value
Gender, F/M	40/45	84/106	23/37	.575
Age at the scan (SD), y	37.3 (11.2)	34.6 (11.6)	33.3 (10.4)	0.076
Duration at the scan (SD), y	18.2 (11.1)	15.2 (11.4)	14.6 (9.7)	0.069
Handedness, Right/Lefe/Ambidextrous	75/9/1	163/22/5	54/6/0	.693
Localization of epilepsy, n, (%)				.755
Temporal	61 (71.7)	134 (70.5)	43 (71.7)	
Frontal	13 (15.3)	34 (17.8)	14 (23.3)	
Parietal	8 (9.4)	15 (7.9)	3 (5)	
Occipital	0	1 (0.3)	0	
Undetermined	3 (3.6)	4 (2.1)	0	
Lateralization of epilepsy, n, (%)				0.621
Left	41 (48.2)	83 (43.7)	30 (50.0)	
Right	40 (47.0)	86 (45.3)	23 (38.3)	
Bilateral	2 (2.4)	14 (7.4)	6 (10.0)	
Undetermined	2 (2.4)	7 (3.6)	1 (1.7)	
History of febrile seizures, n, (%)	6 (7.0)	19 (10.0)	5 (8.3)	0.720
Hippocampal sclerosis, n, (%)	14 (16.4)	39 (20.5)	15 (25.0)	0.450
Seizure frequency				0.582
Less than once a month, n, (%)	11 (12.9)	17 (8.9)	4 (6.7)	
Monthly to weekly	18 (21.2)	57 (30)	15 (25.0)	
Weekly to daily	38 (44.7)	71 (37.4)	25 (41.7)	
Daily seizures	18 (21.2)	45 (23.7)	16 (26.6)	
History of GTCS, n, (%)	54 (63.5)	127 (66.8)	39 (65.0)	0.860
Scanner, original/upgrade	35/50	81/109	21/39	0.576

Abbreviations: ASM: anti-seizure medication; GTCS; generalized tonic clonic seizures; PSM: propensity score matching; SD: standard deviation

Supplementary Table 3. Drug load difference between excluded and included candidates on "moderate" ASMs

	Included	Excluded	P valuce
Monotherapy drug load score	6.5 (1.0-12.0), n=60	5.0 (1.00-12.0), n=53	0.114
Dualtherapy drug load score	13.00(2.5-24.0), n=60	12.0(4.00-23.5), n=87	0.152
Triple therapy drug load score	18.5(7.00-35.0), n=60	16.0 (9.0-35.5)n=47	0.133

Supplementary Figure 1. The distribution of drug load across mono, dual and triple therapy groups with AMSs with moderate cognitive side effects

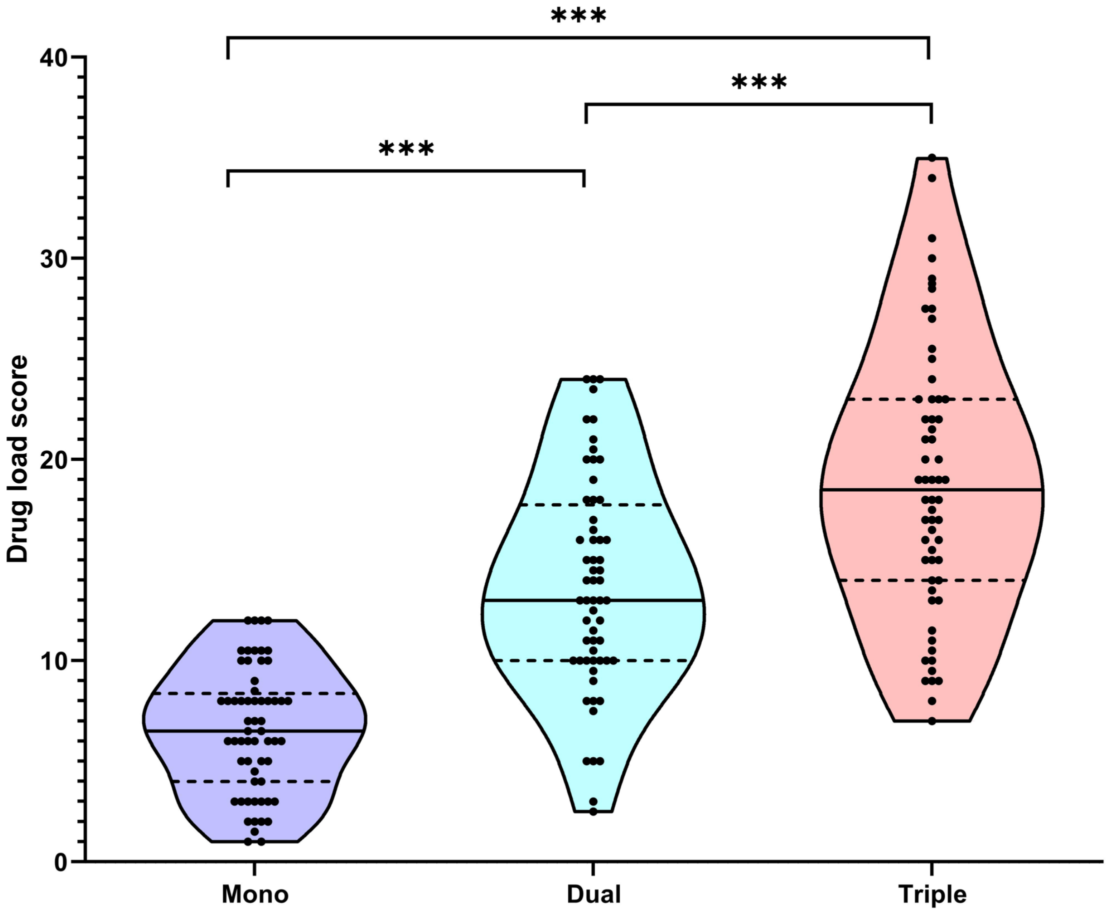

There is significant difference across three groups at drug load scores (F=82.05, P<0.001). The drug load increased with the increase of drug number. The asterisks (***) are post-hoc analyses at P < 0.001, Bonferroni-corrected .

Supplementary Figure 2 Functional activation in language areas and piriform cortex correlates with drug load of ASMs with moderate cognitive side effects after removing the outliers

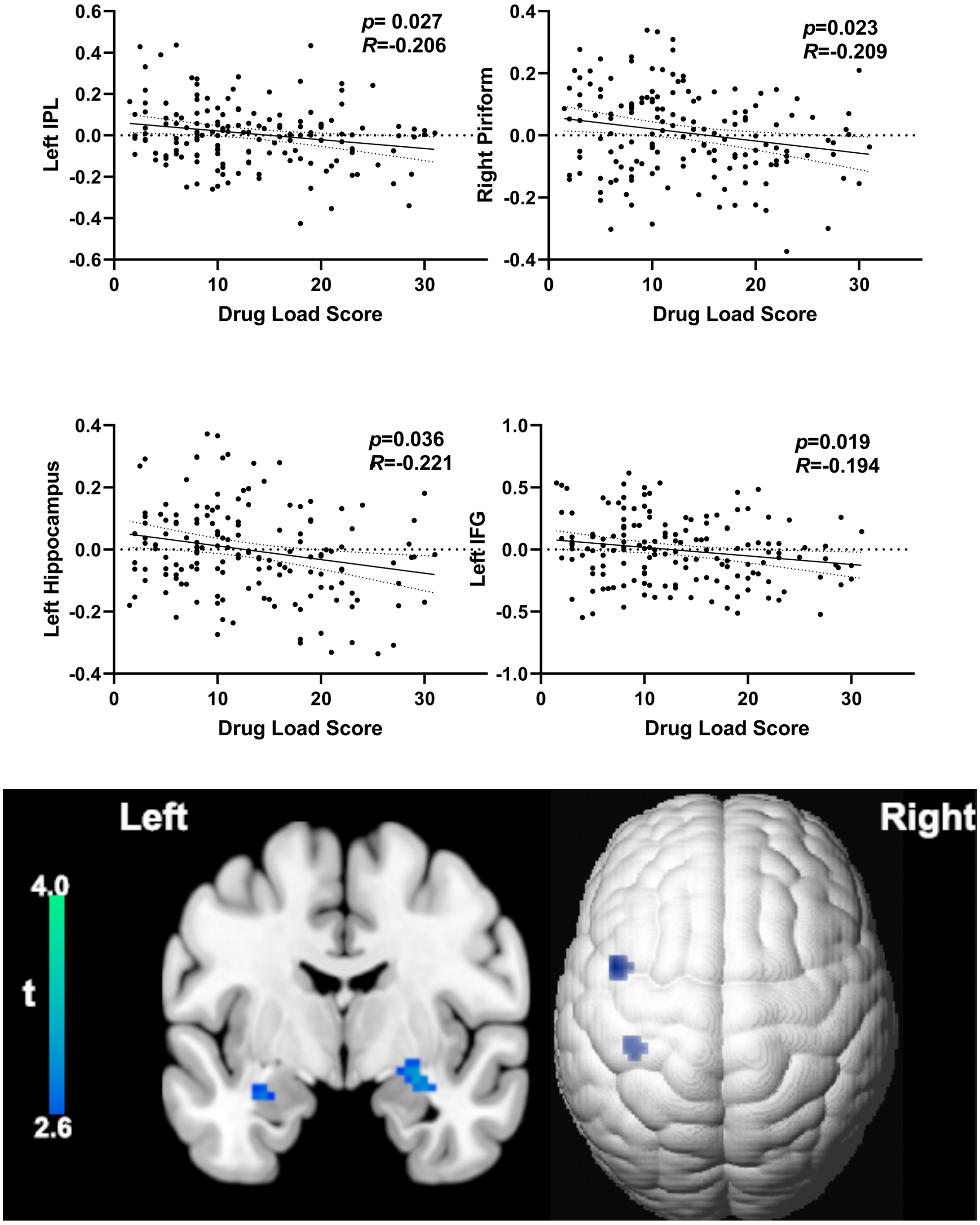

Maps for effects of drug load show voxels included in the 12-mm diameter sphere centred on the local maximum, where peak-level significance at $P<0.05$, FWE-corrected, corresponding a t-score > 2.62.

Supplementary Figure 3 The activation and deactivation patterns of people on monotherapy of anti-seizure medications (ASMs) with moderate cognitive SEs

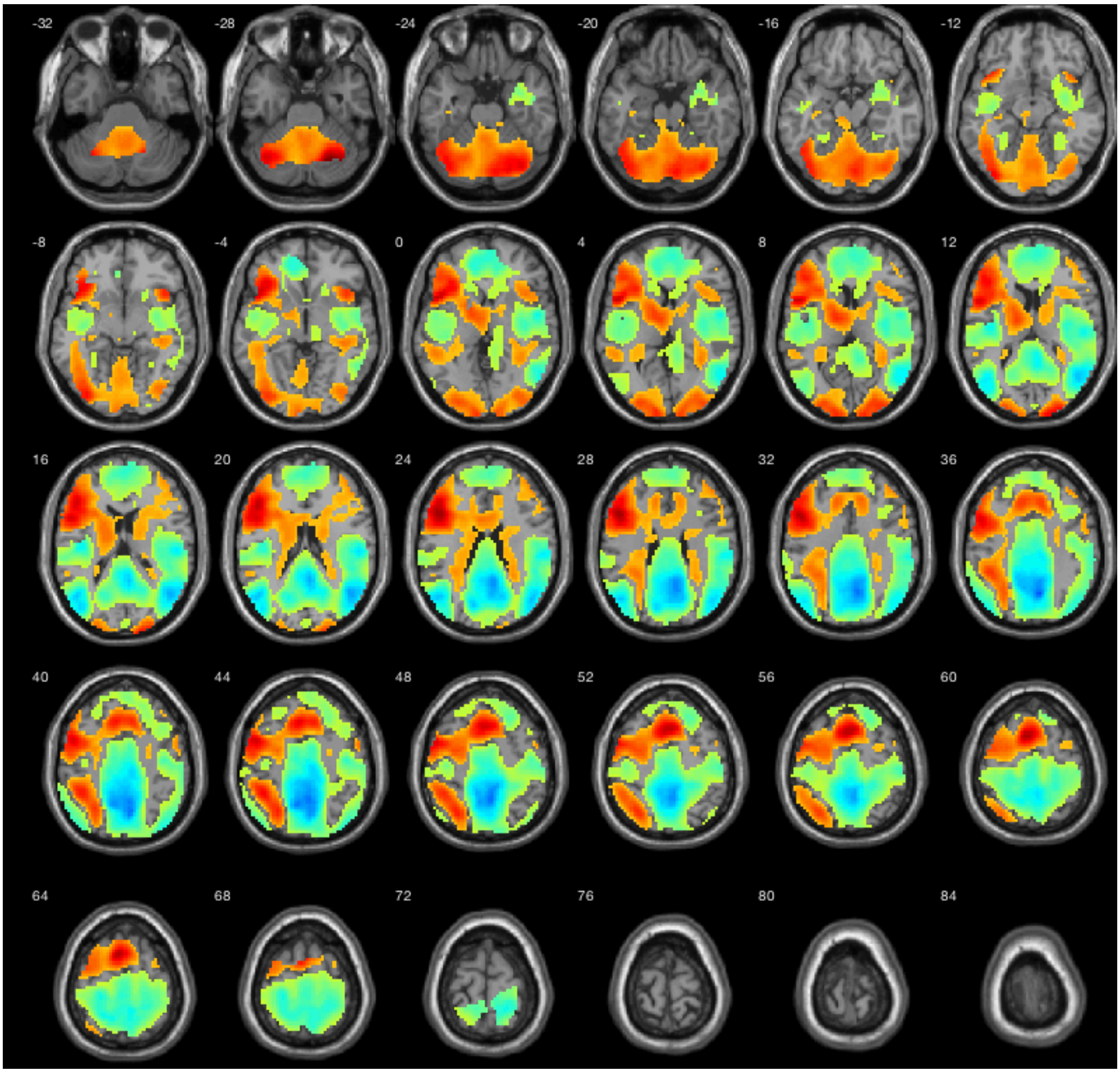

Cortical and subcortical changes are demonstrated superimposed on MNI 152 template. The red stands for activation and the blue stands for deactivation. The threshold is at $P < 0.05$ unccorrected. The right piriform is in the deactivation map.

Supplementary Figure 4 The activation and deactivation patterns of people on dual-therapy of anti-seizure medications (ASMs) with moderate cognitive SEs

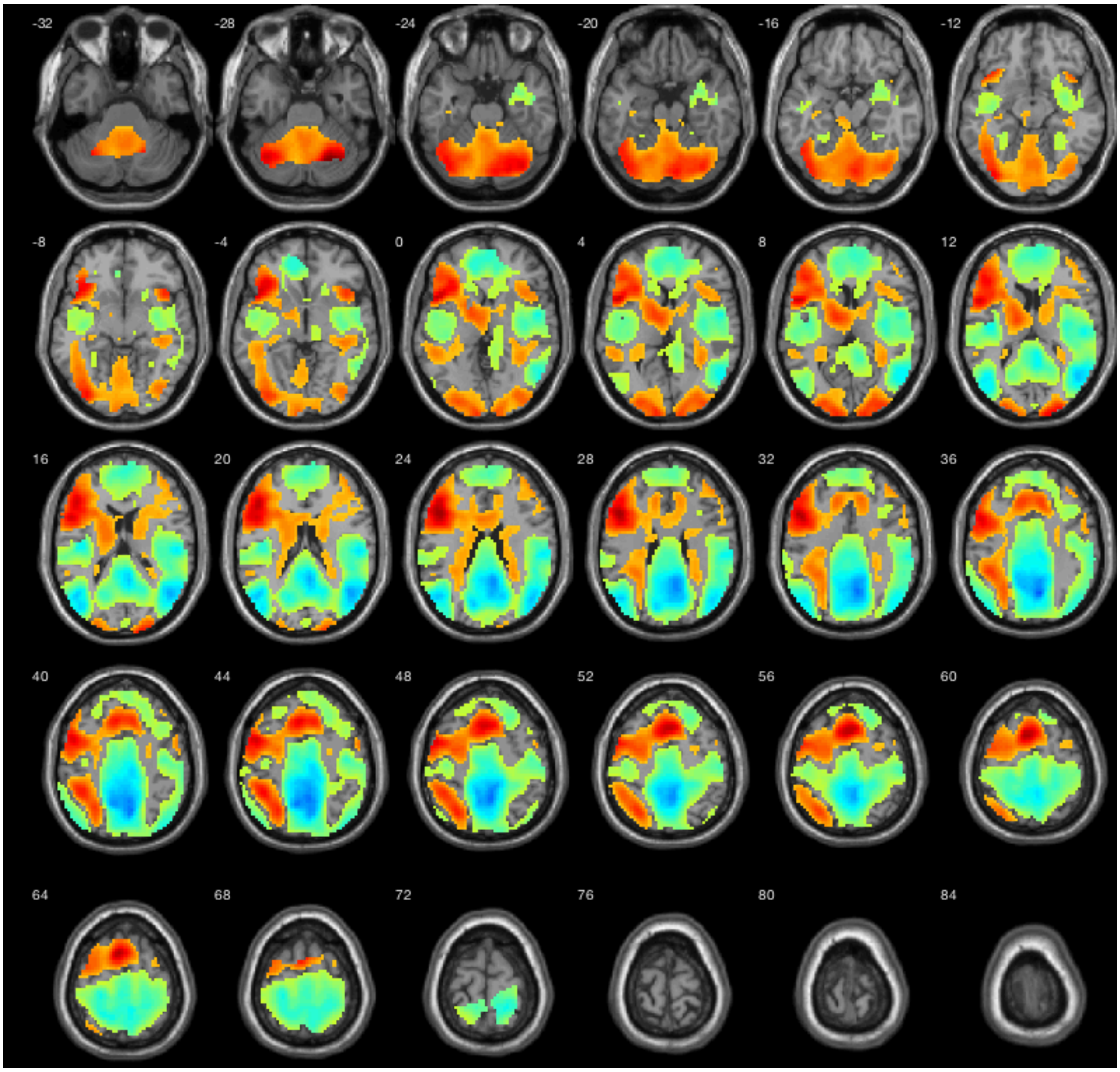

Cortical and subcortical changes are demonstrated superimposed on MNI 152 template. The red stands for activation and the blue stands for deactivation. The threshold is at $P < 0.05$ unccorrected. The right piriform is in the deactivation map.

Supplementary Figure 5 The activation and deactivation patterns of people on triple-therapy of anti-seizure medications (ASMs) with moderate cognitive SEs

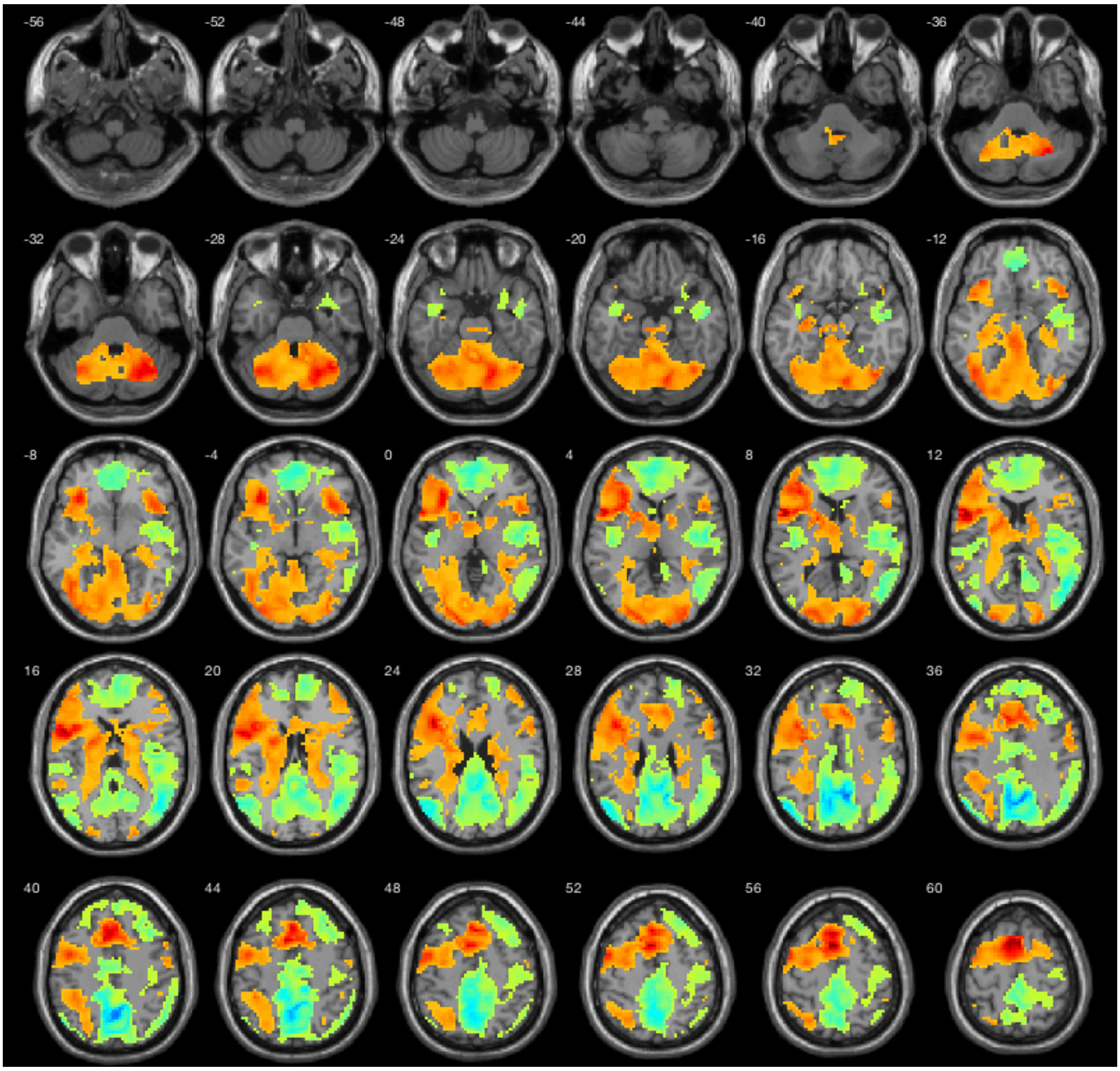

Cortical and subcortical changes are demonstrated superimposed on MNI 152 template. The red stands for activation and the blue stands for deactivation. The threshold is at $P < 0.05$ unccorrected. The right piriform is in the deactivation map.

Supplementary Figure 6 Comparison of VF fMRI between healthy controls (n=62) and patients with “severe” (n=60) and “moderate” (n=60) ASMs group

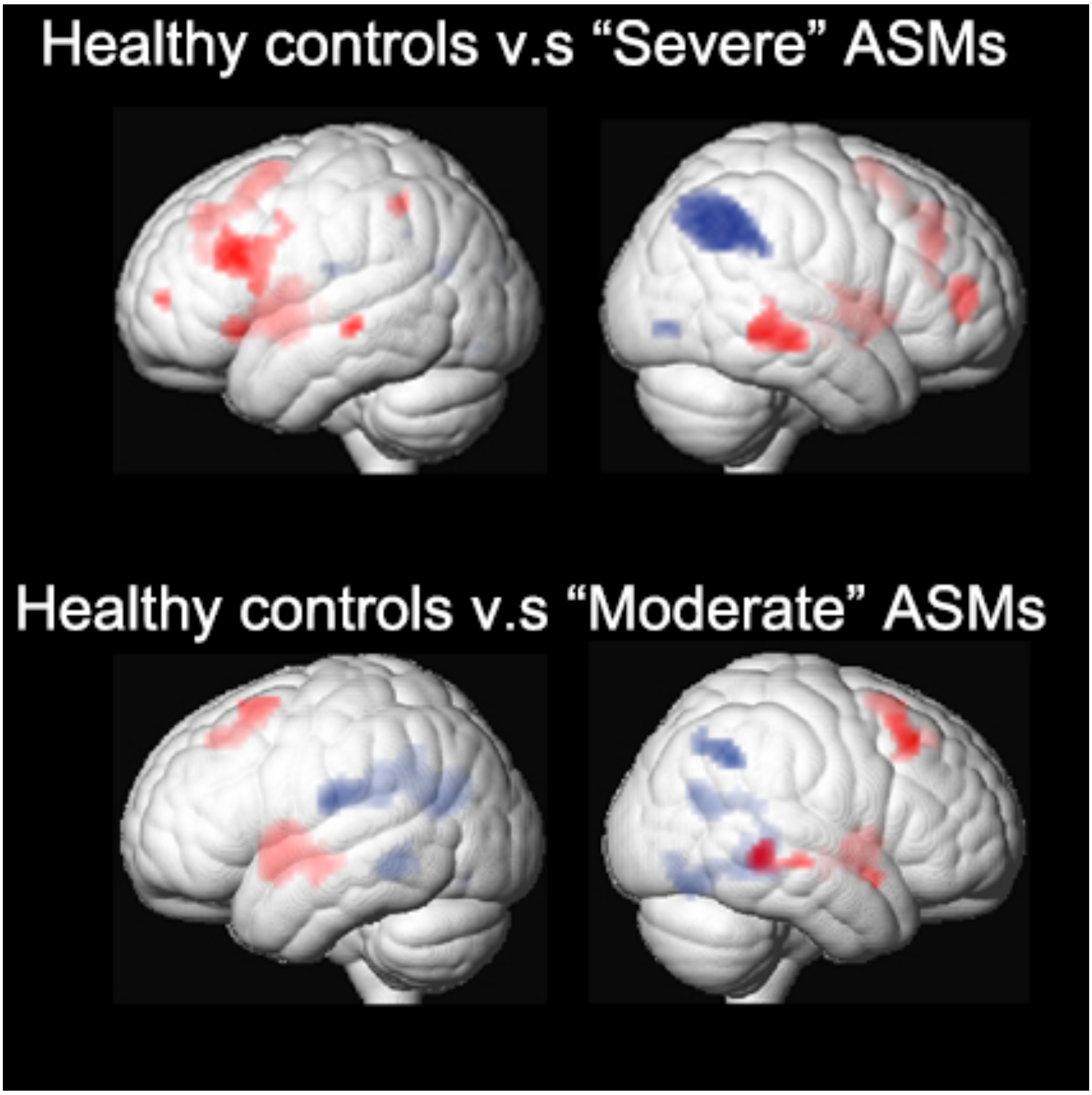

Healthy controls show increased activations in bilateral middle and infeiro frontal gyri and lateral temporal areas and more deactivation of dorsal parietal regions of precuneus. Red: activation; Blue: deactivation; P < 0.005, uncorrected, extent threshold 20 voxels.

Supplementary Figure 7 Comparison of VF fMRI between those who had both fMRI and cognitive tests data

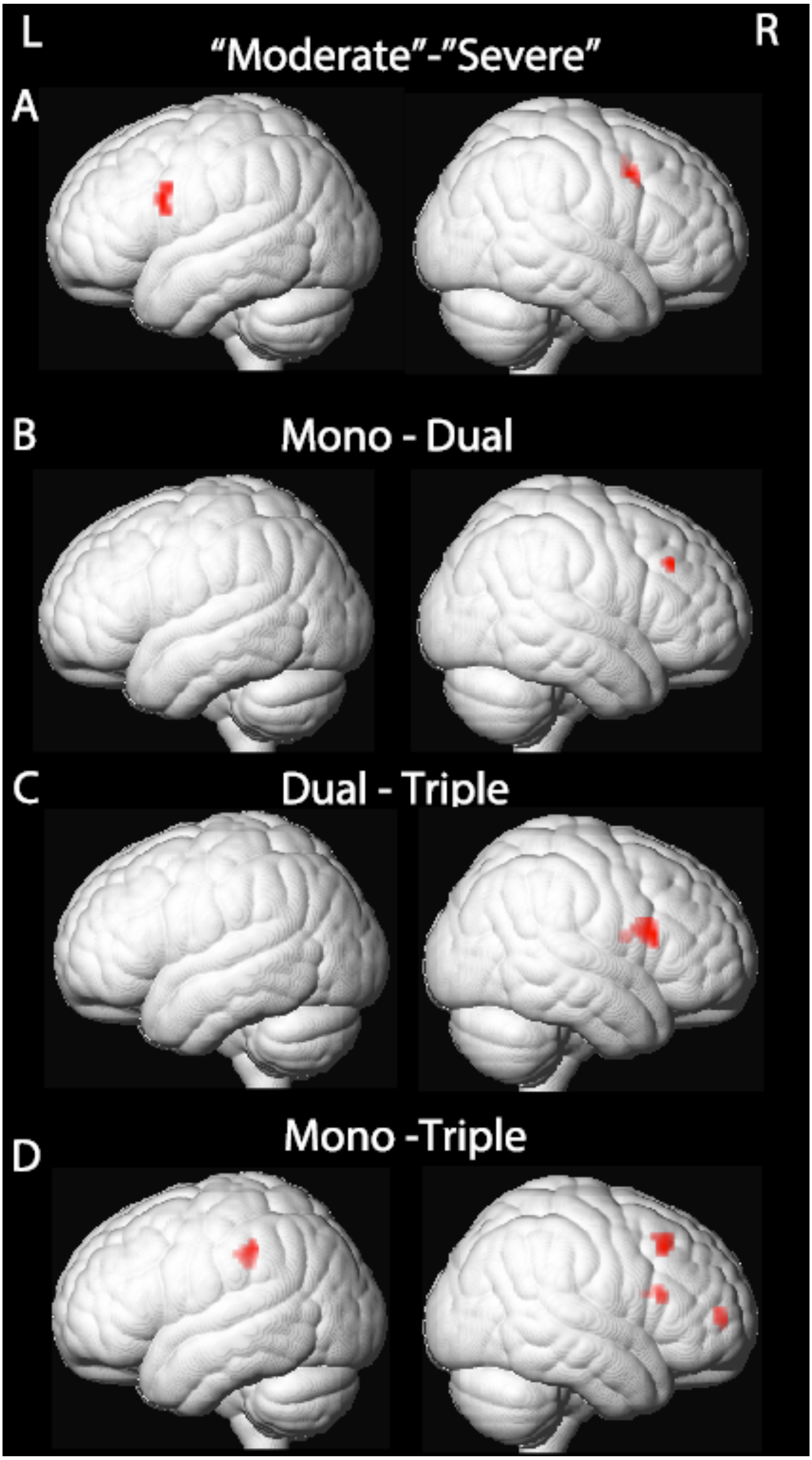

A. Comparison between dual-therapy of ASMs with “moderate” cognitive SEs (n=71) and “severe” cognitive SEs (n=68); B. Comparison between monotherapy (n=40) and dual-therapy (n=36) of ASMs with “moderate” cognitive SEs; C. Comparison between dual-therapy (n=36) and triple-therapy (n=42) of ASMs with “moderate” cognitive SEs; D. Comparison between mono-therapy (n=40) and triple-therapy (n=42) of ASMs with “moderate” cognitive SEs; $P < 0.005$, uncorrected, extent threshold 20 voxels.
